# Clinical Application of a Bird-Beak-Type Z-Shaped Asymmetrical Flap in the Reconstruction of the Inner Canthus

**DOI:** 10.3389/fsurg.2022.786370

**Published:** 2022-08-12

**Authors:** Ze-Chun Huang, Dan Yan, Li-Fang Huang, Hao-Yan Yang, Bin He, An-Li Zhang, Shuai-Hua Li

**Affiliations:** ^1^ Department of Cosmetic and Plastic Surgery, The First People’s Hospital of Chenzhou, Chenzhou, China; ^2^ School of Basic Medicine, Naval Medical University, Shanghai, China; ^3^ Department of Plastic Surgery, Ning Xiang People’s Hospital, Ning Xiang, China; ^4^ Mylike Medical Cosmetic Hospital, Changsha, China

**Keywords:** z-shaped asymmetric flap, overlarge inner canthus, reconstruction, new surgical method, tissue loss

## Abstract

**Objective:**

To introduce a new surgical method for the repair of a large inner canthus combined with tissue loss at the inner canthal angle of the eye by using a bird-beak-type z-shaped asymmetrical flap and to summarize its clinical effect.

**Method:**

A total of 56 patients with a large inner canthus were randomly selected, and a bird-beak-type z-shaped asymmetrical flap was used on the nasal side of the lower eyelid to repair and reconstruct the inner canthal folds. The inner canthal point was located according to physiological aesthetics. The short and long arms of the z-shaped asymmetrical flap were separated, replaced, fixed, and shaped to reconstruct the skin folds of the inner canthus and restore its aesthetic morphology.

**Results:**

All incisions after surgery achieved primary healing, and all 56 cases were followed up for 6–20 months (average 8.6 months). The caruncula lacrimalis was moderately exposed, the inner canthal angles possessed a natural appearance, and the results of the surgery were satisfactory. Five patients developed scar hyperplasia within one month after surgery, and arnica gel was applied topically for 3–6 months until the scar faded or disappeared, but no obvious scars were seen in the surgical area of the remaining patients. In two patients, the internal canthi were asymmetrical, but this improved after adjustment.

**Conclusion:**

Repair of a large inner canthus and tissue loss at the inner canthal angle of the eye using a bird-beak-type z-shaped asymmetrical flap is a simple operation, resulting in minimal trauma. Postoperatively, the inner canthal angle possessed a natural appearance with no obvious scarring.

## Introduction

The epicanthus, also known as the Mongolian canthal fold, is the fold of skin in the medial orbit of the human upper eyelid and is commonly seen in East and Southeast Asian nations, the Khoisan people of South Africa, and the people of Madagascar. It is present in approximately 50 percent of the Asian population. In patients with epicanthus, the normal medial canthus is covered, the palpebral fissure is shortened, and the distance between the two inner canthi becomes wider, which may influence the appearance of the eyes (1). In recent years, many people in China who wish to alter the appearance of their eyes have undergone epicanthus correction. Due to varying surgical methods (2) and variations in skill between surgeons (3), the iatrogenic epicanthus is often overcorrected (4), resulting in deformity of the inner canthus and an unsatisfactory result. In these cases, the caruncula lacrimalis is excessively exposed and the inner canthal angle is enlarged and locally indented downward, with obvious contracture and scar adhesion, resulting in the appearance of angular corners between the scar and the peripheral skin. As a result, the eyes have an aged appearance and patients suffer from problems with dryness and sensitivity to wind and light.

There is very little literature on the complications associated with overcorrection of epicanthus repair, and most of it suggests backtracking the original route in these cases. Although some scholars report the use of a VY advancement flap, the treatment is not satisfactory and can result in incomplete correction and obvious scarring. Most notably, for patients with a large inner canthus and depressed deformity, there is no satisfactory repair method. Therefore, the author performed surgery utilizing a bird-beak-type z-shaped asymmetrical flap on the lower eyelid and nasal side to repair and reconstruct the inner canthal folds. According to the physiological aesthetics of the inner canthus, the proposed inner canthal points were located. The short and long arms of the z-shaped asymmetrical flap were separated, replaced, fixed, and shaped to reconstruct the skin folds of the inner canthus and restore its aesthetic morphology. From February 2018 to October 2019, 56 patients with a large internal canthus were randomly selected, and this surgery was performed to repair the issue. Of these patients, 19 were followed up for more than 12 months, and the clinical data were summarized to evaluate the curative effect.

## Materials and Methods

### Clinical Data

A series of 56 female patients aged 22–45 years were dissatisfied with the results of previous epicanthus correction or repair. Of these patients, 21 cases were not repaired, and 35 cases had undergone one or more epicanthus repairs within 6 months to 2 years (29 cases were repaired once, 4 cases were repaired twice, and 2 cases were repaired three times). The main issues of previous epicanthus correction or repair included a large opening, tissue loss, and depressed scars. The criteria for the malformation of the inner canthus were as follows: mild: iatrogenic hyperplasia of the epicanthus, complete exposure of the lacrimal cartonum, and loss of an aesthetic canthus; moderate: an obvious inner canthal scar, a tissue defect between the inner canthal angle and the lower eyelid margin, and a mild depression deformity; severe: severe depression deformity, separation of the inner eyelid or retraction of the lower eyelid, and associated eye disease (dry eye, conjunctivitis). This study was approved by the institutional research ethics committee of the First People’s Hospital of Chenzhou, and informed written consents were signed by all patients.

### Procedure

The indications for surgery were as follows: a complete exposure of the lacrimal cartonum, an obvious inner canthal scar, a depression deformity between the inner canthus and the lower eyelid margin, a separation of the inner eyelid or retraction of the lower eyelid, and associated eye disease (dry eye, conjunctivitis).

**Figure 1:**
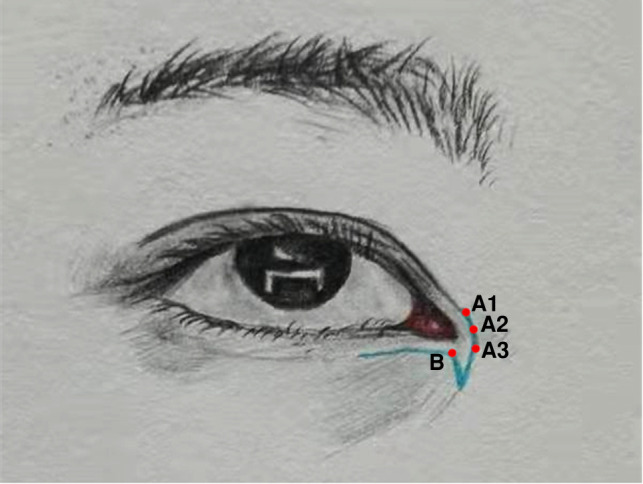
Schematic diagram of the unequal z-shaped flap.

In the skin or scar tissue of the nasal sides and inner canthi, the long and short arms of the z-shaped asymmetrical flap were designed, and the locations of the skin incisions were marked (see Figure 1). Point B was designed as the new inner canthal point, according to ocular aesthetics. The long arm ran 2 mm below the lower eyelid margin and could be extended to the outside of the eye when needed. The short arm was set at the scar ridge on the inner canthus and nasal side. Point A, at the end of the short arm (point A1, A2, and A3; see Figure 1), could be extended to the fold line of the double fold of the upper eyelid, according to the size of the skin folds of the reconstructed inner canthus.

**Figure 2:**
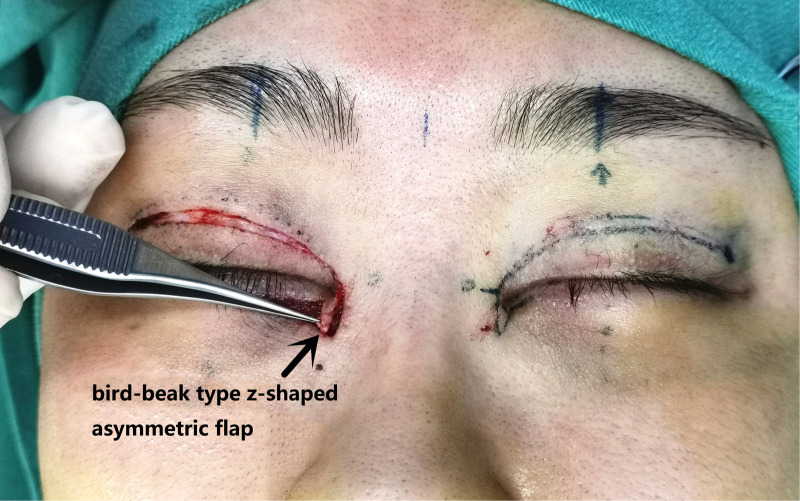
Diagram of the z-shaped asymmetric flap design.

**Figure 3:**
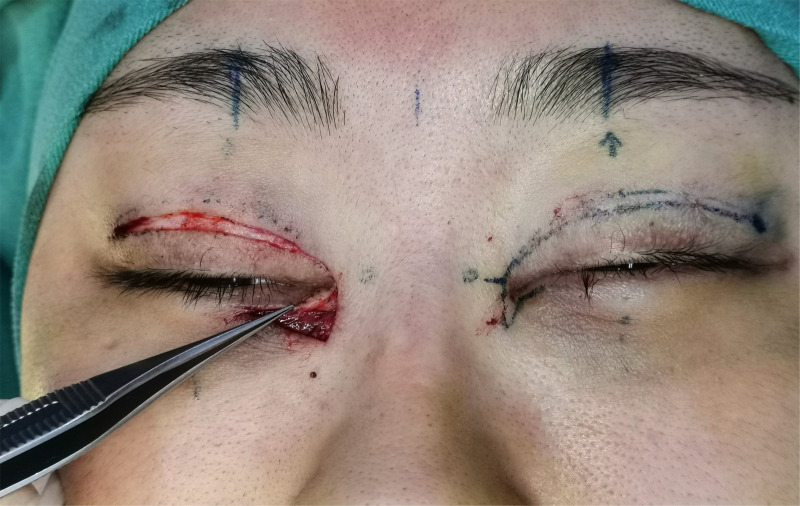
Short-arm scar fascia flap turned 180 degrees upward as the lining of the inner canthus.

**Figure 4:**
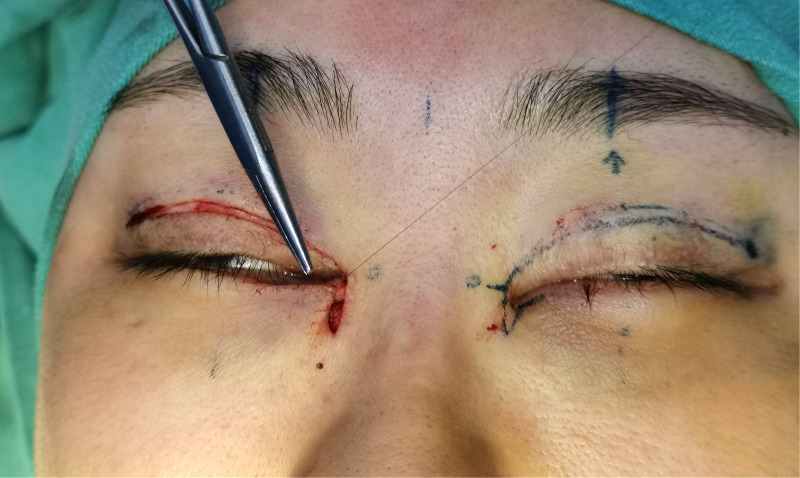
Suture point B on the long-arm flap with point A on the short-arm flap in a suitable position.

Surgical procedure: An incision was made along the designated line and separated in the deep subcutaneous fascial or superficial layer of the orbicularis oculi muscle. The subcutaneous adhesive tissue was fully loosened, and the superficial head of the medial canthus tendon was loosened or partially cut and separated 5–10 mm from the long and short arms of the z-shaped asymmetrical flap incision to the innermost and inferior sides. The short arm of the scar fascial flap was turned upward 180 degrees to create the lining of the inner canthus (see Figures 2 and 3). Absorbable thread (8-0) was used to suture the incision between the upper and lower eyelid margin (two stitches). The z-shaped asymmetrical flap was then set as the covering surface of the reconstructed medial canthal fold, with the medial canthal tendon complex as the fulcrum at the base of the flap and suture point B on the long arm, with point A in an appropriate position on the short arm (see Figure 4). The subcutaneous fascia was then sutured with 6-0 thread (one to two stitches) to reduce tension. If there was a significant loss of subcutaneous muscle fascia on the medial and inferior sides, a narrow orbicularis oculi muscle flap was cut from the inner side of the upper eyelid to fill the area, or retroauricular fascia was used for free transplantation to fill the depressed eye platform. Finally, the skin was sutured with 7-0 nylon thread. The reconstructed inner canthal folds were then observed to ensure they were symmetrical and to assess blood supply to the flaps. The surgical area was then covered with gauze, which was fixed in place with 3M tape to reduce tension. The dressing was changed on the second day after surgery, and the sutures were removed on the seventh day postoperatively.

### Data Collection

Quantitative evaluation of scarring was performed using the Patient and Observer Scar Assessment Scale (POSAS) and Visual Analogue Scale/Score (VAS) by the patient and the plastic surgeon at six months postoperatively. The POSAS is composed of the Patient Scar Assessment Scale (PSAS) and the Observer Scar Assessment Scale (OSAS). The recording parameters of PSAS include color, softness, thickness, concavity, itching, and pain. The recording parameters of OSAS include vascular condition, pigmentation degree, softness, thickness, and concavity. Each parameter was evaluated with a score of 1 to 10, with a lower score representing a superior scar (1 = normal skin; 10 = severe scarring). The VAS was determined by a questionnaire (0 = most satisfied; 10 = most unsatisfied).

The Lacrimal Caruncle Exposure Score (LCES) was evaluated by three plastic surgeons. A score of 0 = completely covered lacrimal caruncle by epicanthus; 1 = less than half of lacrimal caruncle exposed; 2 = over half of lacrimal caruncle exposed; 3 = complete exposure of lacrimal caruncle.

### Statistical Analysis

The SPSS 22.0 software program was used for statistical analysis. Fisher's exact test was used to compare discrete variables, Student's t-test was used to compare continuous variables, and α = 0.05 was used as the test standard.

#### Typical Cases

Case 1: The repair of a deformity caused by the overcorrection of a large epicanthus

**Figure 5:**
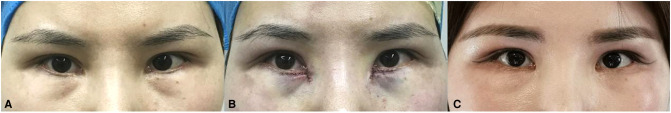
Case 1 (**A**) Before surgery, (**B**) Immediately after surgery and (**C**) 2 years after surgery.

The patient was a 31-year-old female with a one-year history of a deformity caused by the overcorrection of an epicanthus. The inner canthus near the inner side of the lower eyelid margin showed obvious scarring and sunken deformity. The lacrimal caruncle was completely exposed without an inner canthus fold. The author used a bird-beak-type z-shaped asymmetrical flap to reconstruct the inner canthal folds, and the flap was transposed to repair the deformity of the inner eyelid margin. The effect of the surgery both immediately and two years post-operatively was satisfactory (see Figure 5).

Case 2: The repair of a large inner canthus that had been previously repaired.

**Figure 6:**
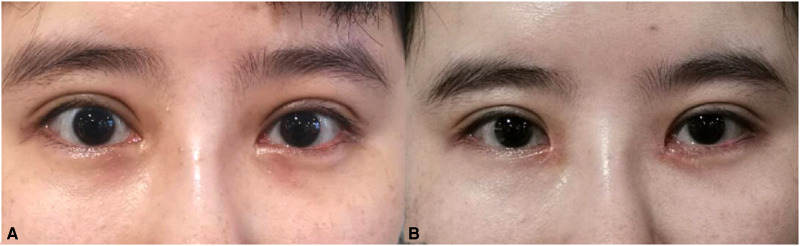
Case 2 (**A**) Before surgery and (**B**) Six months after surgery.

The patient was a 27-year-old female who had undergone a repair due to the poor appearance of the epicanthus one year prior to seeking treatment at our hospital. After her previous epicanthus corrective surgery, the wide inner canthal distance was not improved, the epicanthus was still large, and a depressed deformity was observed in the angular region. The author used a bird-beak-type z-shaped asymmetrical flap to repair and reconstruct the inner canthus fold and the depressed deformity in the angular region. The postoperative results were satisfactory (see Figure 6).

## Results

### General Condition

In this group, 56 patients were followed up for 6–20 months (average 8.6 months). Five patients developed scar hyperplasia within one month of surgery, and arnica gel was applied topically to the local area. The scars faded or became invisible after 3–6 months. No obvious scarring occurred in the other patients. In two patients, the internal canthi were not symmetrical, but this improved after adjustment. In all patients, the caruncula lacrimalis was moderately exposed, the inner canthal angles possessed a natural texture, the inner canthal distance increased by an average of 1.86 mm, and the results of the surgery were satisfactory.

### Results of the Quantitative Evaluation of Scarring at the Six-Month Follow up

On both the POSAS and the VAS, the postoperative and observer scar evaluation scores were significantly lower postoperatively than preoperatively (*P* < 0.05) (Table 1).

**Table 1:** Quantitative score of scar before and after surgery (*n* = 56 cases).

	Before surgery (x ± s)	After surgery (x ± s)	*P*
PSAS score	48.50 ± 3.60	17.15 ± 1.65	0.025
OSAS score	33.64 ± 2.28	12.66 ± 1.23	0.032
VAS score of patients	7.55 ± 0.58	2.85 ± 0.19	0.027
VAS score of observer	6.34 ± 0.36	2.62 ± 0.22	0.012

*The differences were statistically significant (P < 0.05);.*

*Note: POSAS stands for Patient and Observer Scar Assessment and is based on the patient scar assessment score (PSAS) and observer scar assessment score (OSAS) designed in accordance with Vancouver scar scale (accurate to 0.01).*

*PASA contains 6 parameters: color, softness, thickness, unevenness, itching, and pain.*

*OSAS contains 5 parameters: vascularization, pigmentation, softness, thickness, and unevenness.*

*All parameters are 10 points, 1 point means normal skin, and 10 points mean severe scars.*

*The VAS score is subjective and approximate value, with a score of 1–10 according to the degree of satisfaction (0 points mean the most satisfactory and 10 points the least satisfactory).*

### The Caruncula Lacrimalis Exposure Rate and Inner Canthal Distance Scores at the Six-Month Follow Up

The preoperative and postoperative scores for caruncula lacrimalis exposure were 2.80 ± 0.32 mm and 1.27 ± 0.18 mm, and the inner canthal distances were 31.2 ± 2.85 mm and 33.7 ± 3.27 mm, respectively (*P* < 0.05) (Tables 2–4). The average increase in inner canthal distance was 1.86 mm. Additionally, none of the 56 patients had the complication of dry eye or conjunctivitis at the six-month follow up.

**Table 2:** Comparison of preoperative and postoperative carunculae lacrimalis exposure score (*n* = 56 cases).

	Before surgery (*n*)	After surgery (*n*)
0	0	0
1	0	41
2	11	15
3	45	0
Average score	2.80 ± 0.32	1.27 ± 0.18

*The difference is statistically significant (P < 0.05).*

*Note: Lacrimal caruncle exposure score (LCES): 0 point (lacrimal caruncle is completely covered by the epicanthus); 1 point (lacrimal caruncle exposure is less than 1/2); 2 points (lacrimal caruncle exposure is greater than 1 /2); 3 points (full exposure of lacrimal caruncle)*.

**Table 3:** Comparison of internal canthal distance before and after surgery (*n* = 56 cases).

	Before surgery (*n*)	After surgery (*n*)
30–35 mm	23	11
35–40 mm	21	28
40–45 mm	11	17
Average distance	31.2 ± 2.85 mm	33.7 ± 3.27 mm

*The difference is statistically significant (P < 0.05).*

**Table 4:** Extension of inner canthus distance.

Extension of inner canthal distance(mm)	*n*
<1 mm	5
1–2 mm	12
2–3 mm	16
>3 mm	3
Average extension	1.86 mm

## Discussion

Classic corrective surgical methods for epicanthus include Mustarde's method, Blair's method, Spaeth's method, Arlt's method, the Z-horizontal method, the VY advancement flap, and the median canthal tendon shorting method (5). The main objective is to perform a z-shaped flap displacement at the inner canthus to increase the amount of skin in the vertical direction of the inner canthus and to relieve vertical tension on the eyelid (6). At present, epicanthus corrective surgery is performed on a wide range of people, but the complex forces acting on the canthus (7) and the influence of certain factors on the dynamic recovery process may lead to iatrogenic overcorrection of the epicanthus (8). For example, lateral incision of the medial canthus tendon weakens the circular effect of the orbicularis oculi muscle at the medial canthus and expands the angle of the inner corner of the medial canthus. If the rotational flap of the inner canthus is too large, the medial canthus moves downward and expands. The different surgical methods, skill level of the surgeons, and variation in the planning and implementation of the surgery can lead to overcorrection.

Foreign scholar, Dr. Chung (9), backtracked the original route to reconstruct the inner canthus fold. The fold line area was separated subcutaneously on the inner side of the lacrimal caruncle to provide for the inner side of the epicanthus, and a tiny flap was formed. However, due to the limited area of the flap, this method was not effective at reducing local scarring. Some scholars, such as Dr. Lee (10), have used the VY advancement flap to reconstruct the inner canthal fold. However, when using this method on a large epicanthus, the skin from the nasal side does not yield a large enough area, leading to a lateral nasal scar. The lack of tissue on the medial edge of the lower eyelid produces a collapsed deformity of the eyelid margin, making it difficult to reconstruct the lower eyelid support platform using the VY advancement flap. In addition, scarring becomes an unavoidable problem for most people, especially when it is combined with double eyelid repair surgery. It is recommended that epicanthus reconstruction be performed 3–6 months later, when the medial canthus scar has softened (11).

At present, there are many challenging questions facing plastic surgeons performing epicanthus repair, such as: In the case of a large epicanthus, is it possible for backtracking the original route and VY advancement flap repair to achieve the desired effect? What is the appropriate method for replenishing the missing tissue that caused the collapsed notch deformity of the lower eyelid margin? Do the medial canthal tendon and orbicularis oculi muscles need to be treated? What is the best method for dealing with scarring of the inner canthus? What method should be used to prevent scar hyperplasia from occurring again? Because the inner canthus contains multiple sets of mimetic muscles with a complicated structure, the changes in the biological stresses of the local tissue may cause a deformity in the corners of the eyes. After overcorrection of the inner canthal epidermis, the adhesions and mechanical structures of the surrounding tissues of the canthus change, including those at the attachment sites of the skin and muscle fascia. Corrective surgery requires an analysis of the aesthetic characteristics of the eyes and face, the distorted body-surface landmarks, the abnormal local anatomy, tissue loss, changes in muscle function around the inner canthal angle, and the effect of scarring on the reconstruction of the inner canthus. During corrective surgery, the balance of local stress must be considered, as well as appearance, aesthetics, and functional recovery. Surgeons should master the essentials of each step of the surgery, and the scope of the surgical intervention should vary from individual to individual to improve the therapeutic effect of the surgery.

Since 2018, the author has been developing the z-shaped asymmetrical flap to repair overcorrection. This method takes into account the causes of the formation of the epicanthus, the changes in the appearance and structure of the epicanthus caused by the overcorrection, the process of reversing the correction of the epicanthus, and the law of tissue changes after overcorrection. According to the physiological aesthetics of the inner canthi, the original inner canthal point can be located, and the short-arm beak triangular flap of the z-shaped asymmetrical flap can be turned to the inside of the lower eyelid to repair the loss of lower eyelid tissue. It can be used as a lining for the reconstruction of the inner canthus and to hide epicanthus scars. Point B of the long-arm detachable skin flap can be sutured with points A1, A2, and A3, according to the size and amplitude of the reconstructed inner canthal folds. The inner canthus-like physiological folds are rebuilt in a dynamic manner, and the natural texture of the canthus of Oriental people can be restored. In addition, the medial canthus tendon complex can serve as a fulcrum for the reduction of skin tension, which can stabilize tension reduction over the long term and maintain the repair effect. For the collapsed eyelid notch deformity on the medial side of the lower eyelid, the VY advancement flap was previously used in surgery for suturing. As the surgery often increases local tension of the inner canthus and enlarges the deformity, the scarring of the inner canthus will increase, and many surgeons fail to achieve a satisfactory outcome.

If the epidermis of the inner canthus is too large, the inner canthal angle will become too big, and residual primary deformity, tissue loss, and flaws may lead to a canthus deformity. Therefore, the author uses musculofascial tissue to fill this area. If there is insufficient musculofascial tissue, the author cuts a narrow strip of the orbicularis muscle flap from the inside of the upper eyelid to fill the area or conducts retroauricular fascial grafting to support the collapsed lower edge of the eyelid, orbital platform, and narrow canthus. Translocation of local skin or muscle flaps can be used to form new canthal folds and increase the distance between the inner canthi, thus reducing the inner canthal angle. This surgery can be performed to solve complications caused by excessive correction of the epicanthus and restore its original texture for patients who desire a more satisfactory surgical outcome.

In conclusion, the correction of an inner canthal deformity is a challenging surgery that tests the aesthetic sensibility, basic plastic surgery techniques, and surgical skill of the surgeon. This study demonstrated that the bird-beak-type z-shaped asymmetrical flap was a new and simple operation for the repair of a large inner canthus and tissue loss at the inner canthal angle with minimal trauma. Postoperatively, the inner canthal angle possessed a natural appearance with no obvious scarring. However, there were some limitations to this study. Since this was a new surgical technique, the number of cases was insufficient. There was no long-term follow up due to privacy issues relating to plastic surgery involving Chinese people. Additionally, there was a lack of comparison among different degrees, such as mild, moderate, and severe.

## Data Availability

The original contributions presented in the study are included in the article/Suplementary Material, further inquiries can be directed to the corresponding author/s.
